# Weight management during pregnancy: a qualitative study of women’s and care providers’ experiences and perspectives

**DOI:** 10.1186/s12884-017-1538-7

**Published:** 2017-10-11

**Authors:** Sara Holton, Christine East, Jane Fisher

**Affiliations:** 1 Jean Hailes Research Unit, Monash University, Level 4/553 St Kilda Road, Melbourne, Victoria 3004 Australia; 2 Maternity Services, Monash Health and Monash University, Monash Medical Centre, 246 Clayton Rd, Clayton, Victoria 3168 Australia

**Keywords:** Obesity, Prenatal care, Australia, Pregnant women, Midwives

## Abstract

**Background:**

Obesity during pregnancy is a serious health problem for women and their children. Despite the high prevalence of high body mass index (BMI) among women of reproductive age in high-income countries, there is insufficient evidence to inform practice and policy about weight management for women with high BMI who are pregnant. The aim of this project was to describe women’s and midwives’ experiences and perspectives of care for weight management during pregnancy in Melbourne, Australia.

**Methods:**

A qualitative study. Semi-structured interviews were conducted with pregnant women and midwives. Transcripts were analysed thematically.

**Results:**

A total of 17 women and 2 midwives were interviewed. Five themes were identified: 1. Reluctance to and difficulties discussing weight and its implications; 2. Barriers to providing appropriate pregnancy care for women with high BMI; 3. Inconsistent weighing practices; 4. Beliefs about the causes of obesity; and 5. Opportunities to assist women to manage their weight. Although most women were satisfied with the pregnancy care they had received, both women and midwives expressed concerns about effective weight management during pregnancy. These included constraints on discussing weight, difficulties accessing appropriate resources and additional support from other health care providers, and inconsistent weighing practices.

**Conclusions:**

The findings suggest that women with high BMI would benefit from additional information and support about weight management prior to conception, during pregnancy, and postnatally.

## Background

In Australia, it is estimated that almost two-thirds (62.8%) of the population aged 18 years and over are overweight or obese [1]. Almost half the women of reproductive age have a body mass index (BMI) of ≥25 kg/m^2^ [2] which is classified by the World Health Organization as overweight or obese, and up to 43% of women who are pregnant are overweight or obese.

Women who are pregnant and have a high BMI are at elevated risk of obstetric complications including gestational diabetes, preeclampsia, caesarean birth and postoperative infections, and they require longer postpartum hospital stays [3], are less likely to initiate and maintain breastfeeding [4], and may be at greater risk of postnatal depression than women of normal BMI [5]. Their babies are also significantly more likely to be large for gestational age and require neonatal intensive care [3, 6]. Pregnancy care for women with a high BMI is therefore complex and resource-intensive, requires specialist skills, and incurs substantial costs to health services and systems.

There is considerable variation in clinicians’ knowledge of obesity in pregnancy and their education, training and skills for obesity counselling. Many have pejorative views about women in this predicament [7, 8]. National and international guidelines for weight management in pregnancy are focused on diet and exercise recommendations, sometimes coupled with specific information about risks to maternal or fetal health [9–11]. Although such guidelines are important in ensuring a consistent approach to weight management during pregnancy, subjective accounts suggest that current weight management practices can be experienced as shaming, lacking in empathy and generally unhelpful [12].

There is increasing evidence that people with high BMI experience stigma and discrimination in their day-to-day lives [13–15] including in health care-settings [16]. Pregnant women with high BMI report feeling humiliation, discomfort and anxiety during medical examinations and fears that gowns and equipment may not accommodate their bodies [12, 14, 17], but less is known about their overall experiences of and satisfaction with pregnancy care. Very little is known about clinicians’ experiences of providing care for women with high BMI in Australia.

Strategies are required to ensure that women with a high BMI receive evidence-informed, effective, acceptable, supportive clinical care that assists them to manage pregnancy weight gain, and that practitioners and health services are able to provide it.

The aim of this project was to describe women’s and midwives’ experiences and perspectives of care for weight management during pregnancy in Melbourne, Australia.

## Methods

### Study design

Qualitative methods are used to answer questions about experience, meaning and perspective, most often from the standpoint of the participant [18]. This study used individual semi-structured interviews, an established qualitative approach when exploring topics about which little is known [19]. Semi-structured interviews were conducted with two groups: 1. Pregnant women with and without a high BMI; and 2. Midwives providing pregnancy care.

### Setting

Participants were recruited from Monash Medical Centre (MMC), a large tertiary teaching hospital in metropolitan Melbourne, Australia. More than 3600 women give birth at the MMC annually, of whom 44% are overweight and 18% are obese [20]. Women who develop complications during pregnancy and require a higher level of pregnancy care, including women with a BMI > 43 kg/m^2^, are also referred to the MMC for specialist care from other primary and secondary level maternity care services. Standard pregnancy care at MMC includes the provision of a table of recommended weight gain during pregnancy (within the pregnancy booklet given to all women attending the MMC for their pregnancy care) and a Monash Health guideline that recommends regular weighing when attending for pregnancy care clinic visits. In addition to the usual standard of care, the hospital provides a specialised clinic for women with high BMI.

### Sample and recruitment

Inclusion criteria for Group 1 participants were to be an English-speaking woman aged more than 18 years, with or without a high BMI and receiving pregnancy care at the MMC. Women were recruited using purposive recruitment strategies, including via flyers and direct approaches from members of the research team in the hospital waiting room. We aimed to recruit a similar number of women who were receiving standard care and care from the specialised clinic. It was anticipated that an approximate sample of 15–20 women would provide sufficient information power for descriptions of different pregnancy care experiences and contribute new knowledge [21].

Inclusion criteria for Group 2 were to be a midwife practising in pregnancy care clinics at MMC. They were invited to participate by researchers during regular staff meetings. As the midwives invited to participate in the study would have specific experiences and knowledge of pregnancy care for women with high BMI, a sample of 3–5 midwives was considered to offer adequate information power [21].

Participants for both groups were recruited between March and November 2016, and interviews were conducted between March 2016 and March 2017.

### Data source

Interview guides were developed, informed by the investigators’ clinical and research expertise, the published literature relating to weight management in pregnancy, and the research questions. The interviews with women in late pregnancy sought their reflections on experiences of receiving pregnancy care as women with and without high BMI, including experiences of stigma or discrimination, beliefs about causes of high BMI, and the most effective ways to assist women manage weight during pregnancy. Women were also asked about their childbearing experiences including their ideal number of children; number of pregnancies, live births and unintended pregnancies; fertility problems, and age at first birth. At the end of the interview, participants were asked for sociodemographic and other information, including age, country of birth, relationship status, highest level of education, health insurance status, and their pre-pregnancy height (cms) and weight (kgs). In the postnatal interview, details about women’s most recent birth experience including mode of delivery, the baby’s weight, and women’s weight at the end of pregnancy were sought.

The semi-structured interview guide for midwives elicited participants’ reflections on their experiences of providing care for pregnant women with and without high BMI, beliefs about causes of high BMI, the most effective ways to assist women to manage weight during pregnancy, and the interaction between optimal care provision and contextual factors. Sociodemographic and employment details sought at the end of the interview included country of birth, number of years practised, and the number of women with high BMI they cared for each year.

### Procedure

Women were invited to participate in a telephone interview in late pregnancy (≥ 28 weeks gestation) and 4–6 weeks after giving birth. Midwives were invited to participate in an in-person or telephone interview. All participants were given printed information in plain language about the study and provided either written consent or audio-recorded verbal consent at the beginning of the interview. All interviews were conducted by the first author, audio-recorded with permission, and transcribed verbatim.

### Data management and analysis

Group 1 participants were invited to choose a pseudonym. Those who did not were assigned one from the list of the top 20 baby girl names in Australia in 2016. Participants were identified in the analysis by pseudonym only. In order to distinguish the midwives from the women, midwives were not given a pseudonym but identified as “Midwife 1” or “Midwife 2”.

Self-reported weight and height were used to calculate BMI according to the World Health Organization’s classification of underweight and normal weight (BMI <18.5–24.9 kg/m^2^), overweight (BMI of 25–29.9 kg/m^2^) and obese (BMI ≥30 kg/m^2^) [22]. Transcripts were analysed using thematic analysis techniques commonly practised in qualitative research [23]. As identified by Braun and Clarke [23] this consisted of six phases. Phases 1 and 2: Transcripts were repeatedly read and re-read, and coded. Phases 3–5: Codes were grouped into meaningful categories that described how participants talked about the topics, including contradictions and exceptions. Themes were created, named and defined in order to explain and interpret the content. The overall “story” identified by the analysis was established. Examples of the identified themes were selected in the final phase (phase 6) and related back to the research objective. The analysis was conducted by the first author and interpretations discussed within the research team until consensus was reached. Quotes have been used in the text to illustrate the findings.

## Results

Interviews were conducted with 17 women and two midwives. Among the pregnant women, 15 chose to be interviewed by telephone, two in person. All women who participated in a pregnancy interview, except for one who could not be contacted, also completed an interview 4–6 weeks after giving birth. The characteristics of the participants are shown in Tables 1 and 2. Most were aged in their early thirties, born outside Australia, had a postsecondary school qualification, and just over half were overweight or obese. Seven of the women were receiving standard pregnancy care whilst nine attended a speciality pregnancy clinic for women with high BMI (>43 kg/m^2^).

**Table 1:** Women’s sociodemographic characteristics and childbearing experiences

Characteristic	Women (*n* = 17)	Women giving birth in Australia^a^
Average age (years) (range)	32.6 (24–43)	30.4
Country of birth		
Born in Australia	7 (41.2%)	67%
Born overseas	10 (58.8%)	33%
Highest level of education		
Post-secondary school qualification	15 (88.2%)	
No post-secondary school qualification	2 (11.8%)	
Relationship status		
Partnered	17 (100%)	
Not partnered	0 (0.0%)	
Healthcare concession card		
Yes	2 (11.8%)	
No	15 (88.2%)	
Private health insurance		
Yes	9 (52.9%)	
No	8 (47.1%)	
BMI		
Normal weight (<24.9 kg/m^2^)	8 (47.1%)	54%
Overweight (25–29.9 kg/m^2^)	3 (17.6%)	26%
Obese (≥30 kg/m^2^)	6 (35.3%)	20%
Fertility problems		
Yes	5 (29.4%)	
No	12 (70.6%)	
Average ideal number of children (range)	2.6 (2–4)	
Average number of pregnancies (range)	2.3 (1–6)	
Average number of live births (range)	0.8 (0–2)	
Average number of unintended pregnancies (range)	0.2 (0–2)	
Average age at first birth (years) (range)	30.0 (19–41)	
Mode of delivery (index pregnancy)		
Vaginal	9 (56.3%)	67%
Caesarean section	7 (43.8%)	33%
Average baby birth weight (kgs) (range)	3.3 (2.4–4.1)	3.335
Baby admitted to NICU or SCU		
Yes	6 (40.0%)	15%
No	9 (60.0%)	
Infant feeding		
Breastfeeding	16 (100%)	96%
Average weight at end of pregnancy (kgs) (range)	93.5 (65–150)^b^	
Average weight gain during pregnancy (kgs) (range)	12.8 (−5.5–23)^b^	

^a^Women giving birth in Australia in 2014 [41]

^b^data unavailable from 4 participants

**Table 2:** Women’s individual weight characteristics

Participant	Pre-pregnancy BMI (kg/m^2^)	WHO weight classification
Isla	21	Normal
Ivy	21	Normal
Matilda	21	Normal
Amelia	22	Normal
Chloe	22	Normal
Olivia	22	Normal
Emily	24	Normal
Isabella	24	Normal
Mia	25	Overweight
Zoe	25	Overweight
Charlotte	27	Overweight
Lily	41	Obese
Grace	43	Obese
Ruby	46	Obese
Sophia	47	Obese
Ella	55	Obese
Evie	58	Obese

Two midwives were interviewed, both by telephone. Both were female midwives who practised mostly in the speciality pregnancy care clinic at the MMC for women with a BMI more than 43 kg/m^2^. Each had practised as a midwife for at least 3 years.

### Key themes

Five themes about women’s and midwives’ experiences and perspectives of pregnancy care for pregnant women with high BMI were identified.

#### Theme 1: Reluctance to and difficulties discussing weight and its implications

The midwives reported that many women with high BMI are reluctant to discuss their weight during pregnancy care appointments. They also commented that it can often be difficult to discuss weight with women and many women become defensive if care providers attempt to discuss their weight. Nevertheless, midwives thought it was important that women were aware of the maternal and fetal implications of high BMI during pregnancy, and tried not to make women feel like they were “attacking” them when providing weight management advice.

*Most women we see with high BMI* [in the pregnancy clinic] *don’t want to discuss the weight issue. (Midwife #1)*

*Midwives don’t want to ‘lecture’ women about their weight, they just want to talk about the implications of high weight for pregnancy and the baby. (Midwife #1)*

*We talk to every women in the booking appointment about their BMI and optimal weight for pregnancy so I take that as an opportunity to determine how receptive they would be to a discussion about diet but I make sure I include it in every appointment* [not just for women with high BMI] *… and make sure women are aware that we are not telling them they need to go on a crazy diet but it is about making small changes … I try not to make them feel like I am attacking them … and that any changes they make will benefit them and their baby … and acknowledge any changes that they make. (Midwife #2)*

*Although it can be frustrating caring for women with high BMI, I don’t want to belittle them in any way … I want to make sure they are supported. (Midwife #2)*

Although most women reported positive pregnancy care experiences, were satisfied with the care they received, and perceived that the response from care providers about their weight was appropriate, they felt that care providers should not “criticise” pregnant women who have a high BMI.

*I think women may be hesitant to bring up the issue of weight with the midwives and doctors so they need to be approachable and provide advice. (Zoe, BMI 25-29.9 kg/m* ^*2*^ *)*

*I think that the midwives and doctors at* [the hospital] *don’t make a huge deal about weight … they don’t emphasise your weight but they do tell you the risks. (Ruby, BMI ≥ 30 kg/m* ^*2*^ *)*

*I feel very insecure about my weight …* [the midwives] *really tried not to mention it or make me feel uncomfortable. (Evie, BMI ≥ 30 kg/m* ^*2*^ *)*

Most women had received some dietary advice and information from their care provider but this was mainly about foods that should be avoided during pregnancy such as those that may contain harmful bacteria such as *Listeria*. However, women, both with and without a high BMI, also wanted their care provider to give them advice and information about managing weight and appropriate weight gain during pregnancy, and have opportunities to discuss these with them.

*I asked my doctor for advice about my weight … I have put on 15kgs and would like to know if this is normal and get some tips about what I should eat. (Ivy, BMI < 24.9 kg/m* ^*2*^ *)*

The two midwives stated that they were often guided by women’s individual level of interest in weight management when determining how much information to provide but felt they needed to be “blunt” with women who were at high risk of maternal and foetal complications such as women with a BMI > 40 kg/m^2^. They also thought it was important to manage the expectations of women with a high BMI about the most appropriate settings for labour and delivery. For example, that home or water births are not usually recommended for women with a high BMI. Midwives also tried to focus on discussing establishing better health habits such as regular exercise and healthy eating but reported becoming frustrated if women did not follow their advice.

*It is frustrating to give women advice and information during pregnancy about healthy eating and then see them in the postnatal ward eating McDonald’s … it feels like you are wasting your time. (Midwife #1)*

The midwives felt that women “normalised” obesity and this was especially true for women attending the speciality pregnancy care clinic for women with high BMI.

#### Theme 2: Barriers to providing appropriate pregnancy care for women with high BMI

Several barriers were identified to providing appropriate pregnancy care for women with a high BMI by the midwives, including a lack of suitable equipment, few resources to assist women manage their weight, and limited formal training for midwives about caring for pregnant women with high BMI.

The midwives reported difficulties sourcing suitable equipment and facilities for women with high BMI given the hospital’s limited supply including wide wheelchairs, large blood pressure cuffs, bariatric beds, and wards with appropriate toilet facilities, and felt this affected their ability to provide effective care. Despite midwives’ concerns about sourcing appropriate equipment, most women reported that the equipment used during pregnancy care, labour and birth was suitable for their needs.

*I didn’t have to worry about my weight …* [the hospital] *had all the equipment I needed and it was appropriate for me … everything was organised before my appointments so I wasn’t inconvenienced. This was much better than other hospitals I have gone to which were not prepared for caring for someone* [of my weight]*. … I didn’t have to apologise for being overweight. (Evie, BMI ≥ 30 kg/m* ^*2*^ *)*

Midwives commented that although they attempted to “treat all women the same” regardless of their weight, some women required or would benefit from additional support particularly from other care providers such as dieticians. However, opportunities for additional support were limited and midwives expressed frustration in not being able to refer women easily, especially those who expressed concern about their weight and wanted to take action for individual appointments or group sessions with dieticians.

Although the midwives felt confident about and well-supported in providing pregnancy care for women with a high BMI, they commented that they had received little or no formal training about the care and management of such women and that they had mostly “learnt on the job”.

#### Theme 3: Inconsistent weight management/weighing practices

Many women had expected to be weighed at every visit especially those who had pregnancy-related conditions such as gestational diabetes. However, both midwives and women described inconsistent weighing practices. Women reported not being weighed at all, weighed only at their first pregnancy care appointment, weighed inconsistently during pregnancy, or weighed at every pregnancy care appointment.

*I have never been weighed in the pregnancy clinic (Olivia, BMI < 24.9 kg/m* ^*2*^ *).*

*I have only been weighed at the pre-pregnancy clinic for women with diabetes. I haven’t been weighed at any of my routine hospital visits. (Zoe, BMI 25-29.9 kg/m* ^*2*^ *)*

*I have been weighed by the midwife at every visit. I’ve also been weighed by my diabetes educator at the hospital. (Sophia, BMI ≥ 30 kg/m* ^*2*^ *)*

Several women commented that whether or not they were weighed appeared to be inconsistent and when they sought clarification from their care provider about whether they should weigh themselves were told it was not necessary.

*I have been weighed twice by the midwives. First when I was diagnosed with diabetes and then at another midwife visit but it seems a bit random. I thought I would be weighed at every visit given that I have gestational diabetes. (Emily, BMI < 24.9 kg/m* ^*2*^ *)*

*I was only weighed at my first clinic visit. I asked the doctor if I should weigh myself and was told not to bother. (Lily, BMI ≥ 30 kg/m* ^*2*^ *)*

Some women who were not weighed regularly by their care providers weighed themselves.

*I haven’t been weighed at any of my pregnancy clinic visits … but I weigh myself at home. (Mia, BMI 25-29.9 kg/m* ^*2*^ *)*

Midwives stated reasons for not weighing women including not always having time (especially if the woman was late to her appointment) as they had to prioritise other care activities, and trying to avoid women feeling that they were focused on their weight.

#### Theme 4: Beliefs about the causes of obesity

Midwives and women expressed consistent views about the causes of obesity, and identified time, cost, and psychological and cultural barriers to women being able to manage their weight effectively during pregnancy. Women reported that fast food, soft drinks, and sedentary lifestyles or a lack of exercise were the main reasons people were overweight. Midwives believed that women with high BMI often ate too much food and the “wrong” types of food, lacked nutritional knowledge and motivation to effectively manage their weight, and were time poor. Women had similar beliefs, as well as suggesting that hormones caused weight gain during pregnancy. Several women also commented that some women “use pregnancy as an excuse to eat” and gained weight during pregnancy due to misperceptions such as “eating for two”.

Although few women reported personal experiences of stigma or discrimination due to their weight, both midwives and women identified negative societal stereotypes about people who are obese. Women felt that “*everyone has an opinion on pregnant women’s weight*” (Charlotte, BMI 25–29.9 kg/m^2^) and pregnant women who have a high BMI often receive negative comments from a range of people including strangers and care providers. Concerns for the health of the baby were often the main focus of such comments with women told that their weight would have detrimental effects for their baby. Women also thought that because women with a high BMI may not look pregnant, especially at the beginning of pregnancy, they were often treated differently to other women who are pregnant.

*People question whether women who are overweight during pregnancy are really pregnant … no-one gives up their seat on the train for them … and people stare. (Lily, BMI ≥ 30 kg/m* ^*2*^ *)*

*When I was in the* [hospital] *waiting room, people looked at me differently … people think that you don’t look after yourself or take care of yourself when you are overweight. (Evie, BMI ≥ 30 kg/m* ^*2*^ *)*

Women also felt that negative societal perceptions and comments about pregnant women who have a high BMI may result in women becoming distressed and eating for emotion regulation or self-soothing. Midwives also believed that women who had a high BMI often lacked self-esteem and self-confidence.

Most women were able to identify a number of strategies to assist with weight management during pregnancy even if they did not implement or practice these themselves. These included eating healthy foods, reducing portion sizes, and regular exercise.

Although most women reported being satisfied with their body weight and shape during pregnancy, several had concerns about the amount of weight they had gained and felt “miserable” or that they had gained too much weight. Some women expressed concern about their ability to manage their weight given their pregnancy, especially in the first trimester if they had morning sickness and struggled to find suitable food to eat.

*I think I gained weight due to severe morning sickness. The only thing I could eat was bread which helped to stop the nausea and heartburn. I ate bread even when I wasn’t hungry as it alleviated the alkaline taste on my tongue. (Ivy, BMI < 24.9 kg/m* ^*2*^ *)*

*I have found it difficult to feel full whilst I have been pregnant so have eaten a lot more than I usually would. (Isabella, BMI < 24.9 kg/m* ^*2*^ *)*

#### Theme 5: Opportunities to assist women manage their weight during pregnancy and postnatally

Women expressed concern about managing their weight during pregnancy and losing excess weight after the birth. Women and midwives identified several possible interventions to assist women manage their weight. They recommended that women with high BMI be offered specialised services and support similar to that which pregnant women with other conditions such as gestational diabetes or women having multiple pregnancies/births currently receive. For example, group information sessions about weight management offered as part of routine pregnancy care supported with printed or web-based information and individualised appointments with allied health care providers as is done with women with gestational diabetes. It was suggested that midwife-led, non-judgemental group sessions would provide a safe place for women to talk and receive information about weight management.

*I saw a dietician at the pre-pregnancy clinic* [due to diabetes]*. She gave me useful information about food groups and healthy eating during pregnancy. I think other women would benefit from similar information. (Zoe, BMI 25-29.9 kg/m* ^*2*^ *)*

*When we have women who are diagnosed with gestational diabetes they have an education session, I think something like that is needed for women with a high BMI … a group session. (Midwife #1)*

*I think the midwives could offer a group information session like what the twin clinic does for women having twins … they could talk about nutrition and engage women with a high BMI in a group … it could be a safe place … where women could ask questions … and where we could also address all the risks of high BMI in labour. (Midwife #2)*

*I think midwives should give women information about managing their weight during pregnancy and this could include pamphlets or free seminars or groups sessions like mothers’ groups. (Emily, BMI < 24.9 kg/m* ^*2*^ *)*

*I think midwives should speak to women at antenatal classes and at individual appointments about managing their weight, particularly if the midwife has concerns about a woman’s weight or weight gain. (Isla, BMI < 24.9 kg/m* ^*2*^ *)*

Women reported positive experiences with online pregnancy support groups, smartphone apps, and web-based tools in seeking and obtaining weight management information. Midwives were identified as the most appropriate people to talk to about managing their pregnancy weight. Women thought that care providers should discuss weight management with women and suggested that women should be given information about healthy eating, a diet plan, and recommendations about appropriate exercise. Women also thought it would be useful for care providers to regularly check women’s weight and that women could also use smartphone apps to track their weight during pregnancy. As well as web-based resources women also identified written information such as pamphlets as useful and thought any information provided should focus on why it is important to manage weight during pregnancy and how to do this, and be framed positively.

During the postnatal interviews participants reported that primary or routine care is mostly focused on the baby not the mother, and it would be useful for care during the postnatal period to include information on the best ways to lose weight. Maternal child and health care nurses who see women regularly after birth in Australia were also perceived as having a role in providing women with information about weight management and losing weight after pregnancy.

Midwives felt that weight management programs or interventions should not just be focused on pregnancy but also on making lifelong changes. Midwives believed that women with high BMI often lacked motivation about making changes during pregnancy so it would be beneficial for programs to be targeted at women prior to pregnancy and given that this may not always be possible then during the postnatal period before future pregnancies.

## Discussion

Being overweight or obese has become more common in countries such as Australia, and the proportion of pregnant women with a high BMI is increasing. Pregnancy care provides an opportunity to assist women to control weight gain and reduce perinatal morbidities. This study provides an important insight into contemporary weight management experiences and perspectives of pregnant women and midwives. Although most women in this study were satisfied with the pregnancy care they had received, both women (with normal and high BMI) and midwives expressed concerns about effective weight management and identified that women would benefit from additional information and support in managing their weight both during pregnancy and postnatally.

Similar to other studies [24–28], we found that both women and midwives find it difficult and are reluctant to discuss weight and weight management during pregnancy care. Having a high BMI is a source of shame and these data suggest that it can be intensified in pregnancy with the awareness that there are risks to the foetus as well as the self. Avoidance is a common (but ineffective) psychological mechanism to reduce embarrassment, awkwardness and shame and it appears that women, especially those with a high BMI, are unlikely to initiate discussion about weight and weight management with pregnancy care providers. Although care providers acknowledge that women need to be informed about the implications of high BMI during pregnancy, it appears that they might avoid or delay discussing weight management because they are unsure about how to communicate effectively without offending, shaming, stigmatising, discouraging, or causing anxiety [29].

As found in other studies [28], most women in this study understood that eating and activity were related to weight but midwives felt that some women lacked the knowledge and skills to effectively manage their weight. Nevertheless, many women expressed dissatisfaction about the amount or lack of information they received from pregnancy care providers about weight management, wanted more weight management information and support during pregnancy and postnatally, and felt that this should be incorporated into routine care. These findings are consistent with others that have highlighted the lack of consistent and reliable information women receive from pregnancy and postnatal care providers about weight management [25, 26, 30].

There is a trend for greater social acceptance of obesity in some social settings [25, 28, 31]. Midwives in this study, similar to those in other studies [24, 28, 31, 32], perceived that women often ‘normalised’ their weight and believed that this may result in less motivation to implement weight management changes measures.

Routine weighing of women at every pregnancy care visit is no longer routine practice in countries such as Australia, the UK and the US. Instead it is recommended that women be weighed at their first pregnancy care appointment and that only women with risk factors for pregnancy complications such as women with high BMI be weighed at every appointment [9, 33, 34]. At Monash Health, however, recognition of the importance of weight management during pregnancy and of Australia’s obesity epidemic has prompted a recommendation to weigh women when they attend for each clinic visit. Nonetheless, weighing practices revealed in this study were inconsistent and many women reported that they had expected to be weighed throughout their pregnancy. This is consistent with other studies where women have expressed a preference for more frequent weighing and believe that it should be part of standard pregnancy care as it would provide reassurance and motivation for weight management in pregnancy and postnatally [26, 35]. Furthermore, pregnancy care providers in a recent Australian study suggested that weighing all women would avoid stigmatising those who are overweight or obese [31].

### Strengths and limitations

This was a small study that recruited participants exclusively from one large teaching hospital located in a metropolitan area. Many of the women were receiving pregnancy care from a speciality clinic for women with high BMI and therefore, their pregnancy care experiences may not reflect those of women with high BMI in general or women receiving standard pregnancy care.

A strength of this study is that is investigated both women’s and midwives’ experiences and perspectives of pregnancy care for women with high BMI assisting to identify possible interventions that will be effective and acceptable to those who use them. Additionally, women who had a BMI within the normal range were also included in the study enabling the perspectives of women of all BMI categories to be considered. This is important given that women with a normal BMI may gain weight after pregnancy and may also benefit from weight management advice and support during pregnancy and prior to their next pregnancy.

Nevertheless, the results are limited by the participation of only two midwives. It was not possible to interview other pregnancy care providers such as obstetricians for this study. Accordingly, the perspectives and experiences of the participants may not reflect those of other pregnancy care providers nor of women and pregnancy care providers in other settings, although similar findings have been found in recent Australian studies [27, 31].

### Implications for health policy and practice

Effective weight management strategies are required in order to reduce the likelihood of maternal and foetal complications, and reduce the risk of future maternal obesity. Pregnancy care provides an opportunity to offer interventions to assist women to manage weight gain and reduce perinatal morbidities.

The findings of this study suggest that effective pregnancy weight management for women with high BMI requires interventions that address the barriers to weight-management during pregnancy, offer clear advice and non-judgemental support, and are provided during both routine pregnancy care (weight-management) and the postnatal period (weight-optimisation prior to conception). Interventions such as these will contribute substantially to enhanced clinical services, and improved weight management, wellbeing, and health outcomes for pregnant women with high BMI.

The reluctance of pregnancy care providers to discuss weight and weight management with women with a high BMI may be due to a lack of confidence in discussing these matters without upsetting women [7, 32]. Taken together, findings of this and other studies [7, 25] suggest that pregnancy care providers may benefit from specific training in counselling women about weight management during pregnancy, and highlight the importance of care providers discussing weight and weight management with women with a high BMI in non-judgemental ways. This will assist in ensuring that women are comfortable and do not perceive their care experiences as humiliating or their care providers as holding pejorative views which may result in women being psychologically harmed, avoiding care, and increasing comfort eating [36].

During pregnancy and the postnatal period, women have regular and frequent consultations with health care providers, and are often motivated to health behavioural changes that may optimise the outcome of their pregnancy [37] and losing gestational weight gain [25]. Australian, UK and US guidelines recommend that weight management during pregnancy requires the input of dieticians or other appropriate health care providers [9, 11, 34]. Many women in this study and others [27] felt that there was currently a lack of specialist nutritional support available to them and believed that support from dieticians during routine pregnancy care would be beneficial to their weight management. Women with gestational diabetes in this study who had consulted a dietician reported positive experiences. This is consistent with the findings of other studies [25, 35] which found that women who were diagnosed with a co-morbidity such as gestational diabetes appreciated the additional support and educational sessions from health care providers including dieticians.

### Future research

There is a lack of research on weight management in women before pregnancy or between pregnancies [38, 39]. Future research in this area would assist in developing interventions to optimise maternal health and weight before future pregnancies.

Previous research has indicated the potential for innovative weight loss interventions that use technology platforms such as Facebook and text messaging in non-pregnant populations [40]. Further research is also needed to investigate the effectiveness of such technologies in weight management during pregnancy.

## Conclusion

As obesity in pregnancy has significant health implications for mothers and their babies, it is important that women receive appropriate and effective pregnancy care. This study indicates that many women would benefit from additional weight management information and support prior to conception, during pregnancy, and postnatally.
